# A reduced exposure heated tobacco product was introduced then abruptly taken off United States shelves: results from a tobacco harm reduction natural experiment

**DOI:** 10.1186/s12954-024-01000-2

**Published:** 2024-04-24

**Authors:** Brendan Noggle, Kevin M. Ball, Andrea Rae Vansickel

**Affiliations:** Center for Research and Technology, Altria Client Services LLC, 601 East Jackson Street, Richmond, VA 23219 USA

## Abstract

**Background:**

A heated tobacco product (HTP) authorized for purchase in the United States by the Food and Drug Administration as a reduced harm product was removed from the market after about 2 years of sales. Adults who used the HTP were surveyed to determine the impact of the introduction and removal of the HTP on past and current tobacco behaviors.

**Methods:**

Adults who were using the HTP before its United States market removal (n = 502) completed a cross-sectional online survey to determine their tobacco use behaviors at three timepoints: prior to HTP initiation, just before HTP market removal, and at the time of the survey which was administered approximately 10 months post-removal. Descriptive statistics summarized outcome variables and paired bivariate testing was used to compare percent change between timepoints. Multivariable logistic regression and general linear models estimated associations of tobacco use behaviors and cigarette consumption.

**Results:**

Overall, significantly fewer adults consumed cigarettes while using HTP than before they tried the product (63.0% vs. 89.9%, *p* value < 0.0001) and the number of cigarettes consumed per week (CPW) decreased (106.3–39.0, *p* value < 0.0001). After HTP removal, the percent of adults who consumed cigarettes increased non-significantly (63.0–67.5%, *p* value = 0.0544) while CPW increased significantly (39.0–76.6 CPW, *p* value < 0.0001). At the time of the survey, over 25% of the sample continued to use the HTP and 7.2% reported use of no tobacco products. Electronic nicotine delivery system use had increased significantly from the prior period (27.4% increase, *p* value < 0.0001).

**Conclusion:**

This study demonstrates reduction or elimination of combustible cigarette smoking while adults were using HTPs and some increased smoking after market removal, albeit at lower levels. If unable to find satisfying alternatives, adults who smoke and transition to reduced harm products may return to smoking or purchase products illicitly if their preferred products are removed from the regulated market.

**Supplementary Information:**

The online version contains supplementary material available at 10.1186/s12954-024-01000-2.

## Introduction

A heated tobacco product (HTP) marketed under the brand name IQOS^®^ was sold in the United States (U.S.) as a reduced exposure alternative to continued smoking [1]. HTPs usually consist of a battery powered device and a processed tobacco consumable. The tobacco is heated at a lower temperature than required for combustion and produces an aerosol containing nicotine and other tobacco constituents that is then inhaled. Many adults who smoke used this HTP as a harm reduction tool and concurrently reduced the amount of conventional cigarettes (CC) smoked or stopped smoking altogether [2]. Approximately 2 years of sales later, this HTP was abruptly removed from the U.S. marketplace without other HTPs legally available for purchase, leaving consumers to decide whether and how to continue using nicotine-containing products [3]. To determine the impact of the introduction and abrupt removal of an HTP on tobacco use behaviors, this survey study compared past, and current tobacco use behaviors and intentions among adults who used the HTP while it was still available for purchase in the U.S.

In April 2019, the Food and Drug Administration (FDA) authorized the sale of the HTP in the U.S. [4] and distribution began in select Southeastern markets by October of the same year [5]. Prior to this, awareness and use of HTPs was low [6]. In 2020, FDA determined that this HTP could be marketed as a reduced-risk tobacco product with a modified risk claim^1^ as a reduced exposure alternative to continued use of cigarettes [1]. The reduced exposure claim was granted on the basis that, while this HTP is not risk-free and contains nicotine, it heats a proprietary tobacco “HTP stick” without combustion, thereby releasing fewer harmful compounds compared to conventional cigarette smoke [1]. Over the following year, HTP distribution grew at a measured pace at physical stores in the Southeast and online. As of November 2021, distribution stopped due to an import ban and a cease-and-desist order issued by the U.S. International Trade Commission (ITC), which restricted the sales, distribution, and marketing in the United States. [3]. However, this HTP (IQOS^®^) [7] and other HTPs (e.g., Ploom, glo, PAX) continue to be sold in other parts of the world [8, 9].

Harm reduction is a foundational practice in public health that seeks to minimize harm from risky behaviors through policies and practices aimed at reducing the health, economic, and social consequences of such behavior while recognizing that stopping certain behaviors is best [10–12]. Harm reduction as it applies to tobacco use remains a controversial practice even though a roadmap exists highlighting how tobacco harm reduction (THR) strategies can reduce or eliminate combustible tobacco use. Resistance to the roadmap is primarily driven by fears of tobacco initiation among current non-users [13, 14]. Health and public health organizations in countries such as the United Kingdom, Canada, and Sweden recommend e-vapor or snus use as a method to reduce or eliminate smoking [15–17]. The U.S. public health organization Centers for Disease Control and Prevention recently noted “e-cigarettes have the potential to benefit adult smokers who are not pregnant if used as a complete substitute for regular cigarettes and other smoked tobacco products” [18] but published, state-based tobacco control strategies have not been updated in a decade and do not mention this type of harm reduction approach [19]. Researchers and authoritative bodies generally agree that most tobacco-related harm comes from exposure to the products of combustion and that non-combustible tobacco consumption carries relatively fewer health risks [12, 20–22]. Thus, switching adults who smoke to a non-combustible alternative such as an HTP would be considered a harm reduction approach and, while it would carry more exposure to harmful constituents than quitting tobacco altogether, it would still provide an opportunity to reduce exposure for those unwilling or unable to completely quit tobacco use. The opportunity for harm reduction exists as recent adult tobacco consumer data demonstrates that almost two in three (63%) adults who smoke are at least somewhat interested in completely switching from smoking to a non-combustible alternative.^2^

While smoking prevalence is decreasing due to decreased initiation and increased cessation, estimates predict that smoking will persist in the near future. Mendez (2022) and colleagues examined trends in smoking incidence and cessation from U.S. nationally representative surveys to estimate future smoking prevalence and predicted adult smoking prevalence would fall to 8.3% around 2030 and eventually reach a steady state of 3.5% [23]. These authors concluded that smoking reduction has accelerated in recent years, likely due to a combination of traditional tobacco control policies and the introduction of new non-combustible smoking alternatives that deliver nicotine [23]. While the reduction in smoking prevalence is encouraging, there remains a population of adults who either cannot or will not quit smoking. It is these adults who smoke that could benefit from harm reduction strategies.

The introduction and then abrupt removal of HTP for purchase in the U.S. provides a rare natural experiment that can be leveraged to understand the impact of reduced harm products on tobacco use and stopping smoking behaviors. In this study, HTP consumers were surveyed on tobacco use behaviors approximately nine to 10 months after HTP market removal to allow time for behaviors to stabilize. We hypothesized that many of the adults who smoked prior to using HTP might revert to prior tobacco use patterns or switch to other noncombustible tobacco products such as e-cigarettes.

## Methods

**Fig. 1:**
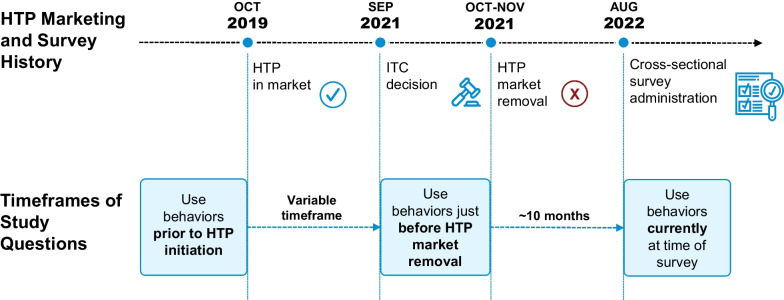
Summary of HTP marketing events in the U.S. and time periods referenced in the study

This online, cross-sectional survey study compared adults’ (21+) tobacco product use behaviors before they initiated use of the HTP, while the HTP was marketed in the United States, and after the HTP was removed from the market. Figure 1 details U.S. HTP marketing and study time periods.

We recruited participants from an IQOS^®^-specific consumer database, which covered an estimated 70% of individuals who purchased the HTP device. Consumers could register their device online to receive discounts or other benefits and agreed to be contacted for future research. Invitations were sent by email to each of the approximately 15,000 individuals in the database who had agreed to be contacted for research purposes and had a valid email address. Of the individuals invited, 1,051 entered screening and 541 were excluded for reasons including exiting or declining screening (49.2%), non-compliance during age verification (20.9%), ever employed or related to someone employed in the tobacco industry (11.6%), non-use of HTPs (10.4%), and other reasons. Of the 510 eligible individuals who consented to participate, 502 (98.4%) completed the survey and were included in the analysis. Participants were English-speaking U.S. resident adults aged 21 years or older and responded that they used the HTP (i.e., IQOS^®^) in the 3 months prior to market removal. All participants provided electronic informed consent and were provided a $20 incentive for completing the survey, which took approximately 11–12 min. The study was approved by Sterling Institutional Review Board (Study ID #: 10143). Data were collected between July 22, 2022 and August 9, 2022.

All participants completed a self-administered online questionnaire with questions designed to characterize patterns of tobacco consumption before initiating use of the HTP, within 30-days prior to removal of the HTP from the market, and in the 30-day period prior to taking the survey (“current use” post-market removal). Tobacco and nicotine product categories included cigarette, the HTP, electronic nicotine delivery system (ENDS), smokeless tobacco, nicotine pouch, some other tobacco product and a stop smoking aid. Wherever feasible, survey items were sourced and/or adapted from U.S. national surveys and cognitively tested items from previous internal studies. Checklist items were randomized. Study measures included:

- Use of tobacco and nicotine product categories: Marked as “Yes” to the question “Which of the following tobacco or nicotine products did you use…” in the past 30 days at each study time period
- Former use: Use of tobacco and nicotine product categories in a prior study time period but not a subsequent study period
- Amount of cigarettes or HTP sticks consumed per week: Estimated by multiplying the number of days per week consumed cigarettes or HTP sticks and number of cigarettes or HTP sticks consumed per day among consumers of each product at each study period
- Smoking cessation: Smoking before initiating use of the HTP or within 30 days prior to removal of the HTP from the market and then no longer smoking in a subsequent study period
- Future use intentions: a fully labelled 6-point Likert-type question with the stem “How likely or unlikely are you to do each of the following in the future?” for each tobacco and nicotine product category with responses ranging from 1 “definitely not likely” to 6 “definitely likely”
- Stockpiling: Marked as “Accumulated a large amount of [HTP sticks] to have on reserve (i.e., Stockpiled [HTP sticks])” through a multiple-choice question “What did you do when you learned that [the HTP] was being removed from the market?”

Descriptive statistics summarized outcome variables. Paired bivariate testing using the McNemar statistic was used to determine statistical significance of proportions between timepoints for categorical variables such as percent “yes”. T-tests were used to determine statistical significance of means between timepoints of continuous outcomes such as cigarettes per week (CPW). Multivariable logistic regression models estimated the association of HTP use, ENDS use, and other tobacco use variables with stopping smoking while accounting for demographic covariates. Race-ethnicity was categorized into four groups: White, Black, Asian (Asian, Indian, Pacific Islander (PI)), and Other (including Multiracial and Hispanic or Latino) in regression models to provide robust estimates and sample sizes. Use of non-cigarette combustible tobacco products was removed from models due to insufficient sample size. Additionally, a general linear model estimated the relationship between the number of cigarettes smoked per week at the time of survey and tobacco use and demographic variables. The data analysis for this paper was generated using SAS software, version 9.4 (SAS Institute, Cary, NC, USA).

## Results

Overall, this study found, use of the HTP was associated with cigarette smoking reduction. Additionally, 9 months after the HTP market removal, amount of cigarette smoking increased but not to original levels and one quarter of participants reported still using the HTP with the HTP sticks, even though U.S. distribution had ceased.

Table 1 shows participant demographic characteristics, which were consistent with previous HTP regulatory studies [2]. Of 502 participants, the mean age was 45 years, included slightly more males than females (54% vs 46%), and 80% identified as white or Caucasian. Annual household income was greater than $100,000 for almost 30% of participants and over one in three had at least a bachelor’s degree; this is generally higher income and education than that of a typical U.S. adult who smokes cigarettes [24, 25]. Almost all HTP consumers lived in the South, which reflected the marketing and distribution area.

**Table 1:** Participant demographic characteristics

Demographic characteristic	n (%)
Age category (years)	
Mean age ± SD	44.7 ± 11.4
21–34	95 (18.9%)
35–49	236 (47.0%)
50–64	141 (28.1%)
65+	30 (6.0%)
Gender	
Male	273 (54.4%)
Female	229 (45.6%)
Race	
White or Caucasian	400 (79.7%)
Black or African American	41 (8.2%)
Multi-racial	11 (2.2%)
Another race	50 (9.9%)
Ethnicity	
Hispanic or Latino	27 (5.4%)
Non-Hispanic or Latino	475 (94.6%)
Geographic Region	
Midwest	2 (0.4%)
Northeast	2 (0.4%)
South	496 (98.8%)
West	2 (0.4%)
Annual Household Income	
Less than $50,000	150 (29.9%)
$50,000 to $100,000	180 (35.8%)
More than $100,000	140 (27.9%)
Declined to answer	32 (6.4%)
Level of education	
High school diploma or less	107 (21.3%)
Some college or Associate degree	217 (43.2%)
Bachelor’s degree or higher	171 (34.1%)
Unknown level of education	7 (1.4%)

**Fig. 2:**
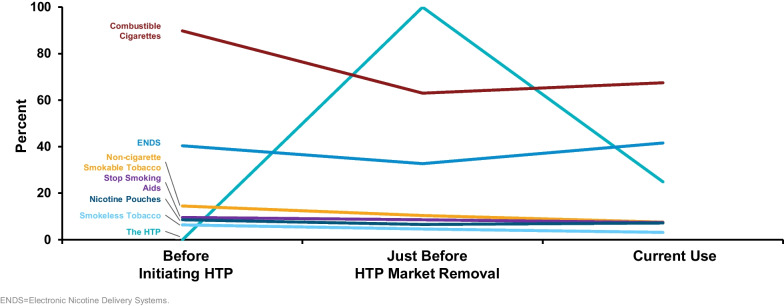
Reported tobacco use percent across three study time periods

Table 2 and Fig. 2 show reported tobacco use across each of the study time periods (prior to HTP initiation, just before HTP market removal, current use) among participants who met the case definition.

**Table 2:** Reported tobacco use and percent change across three study time periods

TNP category	Prior to HTP initiation	Just before HTP market removal	Current use^1^	Use prior to initiation versus use just before market removal		Use just before market removal versus current use		Use prior to initiation versus current use	
	% (n)	% (n)	% (n)	% Change	*p* value	% Change	*p* value	% Change	*p* value
Combustible Cigarettes (CC)	89.8% (n = 451)	63.0% (n = 316)	67.5% (n = 339)	− **29.9%**	***p* < 0.0001**	7.30%	*p* = 0.0544	− **24.8%**	***p* < 0.0001**
HTP^2^	0.0% (n = 0)	100% (n = 502)	24.9% (n = 125)	–	–	− 75.1%	–	–	–
Electronic Nicotine Delivery Systems (ENDS)^2^	40.4% (n = 203)	32.7% (n = 164)	41.6% (n = 209)	− **19.2%**	***p* = 0.0005**	**27.4%**	***p* < 0.0001**	3.0%	*p* = 0.6069
Smokeless Tobacco Products^2^ (STP)	6.4% (n = 32)	4.6% (n = 23)	3.2% (n = 16)	− **28.1%**	***p* = 0.0290**	− 30.4%	*p* = 0.0896	− **50.0%**	***p* < 0.0001**
Nicotine Pouches^2^ (NP)	8.6% (n = 43)	6.6% (n = 33)	7.2% (n = 36)	− 23.3%	*p* = 0.077	9.1%	*p* = 0.5775	− 16.3%	*p* = 0.2498
Non-Cigarette Smokable Tobacco Products^2^	14.5% (n = 73)	10.4% (n = 52)	7.6% (n = 38)	− **28.8%**	***p* = 0.0014**	− **26.9%**	***p* = 0.0082**	− **47.9%**	***p* < 0.0001**
None of the Above Products	3.8% (n = 19)	0.0% (n = 0)	7.2% (n = 36)	− 100%	–	–	–	**89.7%**	***p* = 0.0052**
Nicotine Replacement Therapies (NRT)^3^	9.6% (n = 48)	8.6% (n = 43)	7.4% (n = 37)	− 10.4%	*p* = 0.4838	− 14.0%	*p* = 0.4461	− 22.9%	*p* = 0.1521

1—Current use is defined as use at least once in the 30-day period prior to the participant taking the survey. 2—HTP use presented in the survey as IQOS^®^ tobacco heating system; ENDS presented in survey as: electronic nicotine delivery systems (ENDS, also known as e-cigs, electronic cigarettes, vaping devices, vape pens, and/or e-liquids); Smokeless Tobacco Products presented in survey as: smokeless tobacco products (moist snuff, chewing tobacco, snus); Nicotine Pouches presented in survey as: nicotine pouches (tobacco-free pouch placed in the mouth); Non-Cigarette Smokable Tobacco Products presented in survey as: some other smokable tobacco product (such as cigars, cigarillos, pipe tobacco, hookah, or water pipe); used none of these tobacco or nicotine products was included if the participant had not used the aforementioned products and, if selected, no other response option could be selected. 3—Use of NRT was asked independently of the list of tobacco or nicotine products described above. Thus, participants who responded that they used any or none of the aforementioned tobacco or nicotine products could report they had or had not used NRT. NRT presented in survey as: Aids to help stop smoking (e.g., Nicorette, NicoDerm CQ). Bold font signifies a statistically significant difference (*p* value < 0.05) between two time periods

### Use prior to HTP initiation

When asked about the tobacco products used just prior to HTP initiation, 89.8% (n = 451) of participants reported combustible cigarettes, 40.4% (n = 203) reported ENDS, and 14.5% (n = 73) reported other non-cigarette smokable tobacco products. Just under 10% used nicotine replacement therapy (NRT), and 3.8% (n = 19) reported using none of the listed tobacco products.

### Use just before HTP removal

When asked about the tobacco products used just before HTP removal, all participants used HTP, either exclusively or with other products. There was directionally less use of all other tobacco product categories while the HTP was on the market compared to before HTP initiation. Significant use prevalence reductions were seen in multiple product categories including cigarette use declining by 29.9% (*p* < 0.0001), ENDS use declining by 19.2% (*p* = 0.0005), smokeless tobacco use declining by 28.1% (*p* = 0.0290), non-cigarette smokable tobacco product use declining by 28.8% (*p* = 0.0014) and dual use of ENDS and cigarettes declining by 46.7% (*p* < 0.0001).

### Current use at time of survey

Inspection of current use behaviors at the time of the survey (9–10 months after HTP had been removed from the marketplace) revealed a 75.1 percentage point decline of adults who reported use of the HTP. Despite the HTP no longer being legally sold or imported into the US, approximately 25% of participants reported current use of the HTP. After the HTP was removed from market, current smoking non-significantly increased by 7.3% (*p* = 0.0544) among participants from 63.0% to 67.5% at the time of the survey. ENDS use increased 27.4% (*p* < 0.0001) to about the same levels as before initiation of the HTP. Non-cigarette smokable tobacco product use declined 26.9% (*p* = 0.0082) and non-use of any of the listed tobacco products increased to 7.4% (*p* = 0.0052).

### Other outcomes

Compared to use status prior to initiation of HTP, current use patterns showed significant declines in adults who reported smoking from 89.8 to 67.5% (*p* < 0.0001), other non-cigarette smokable tobacco product use decreased from 14.5 to 7.6% (*p* < 0.0001), and tobacco product non-use doubled. About 34% (71 of 209) of adults who reported current ENDS use at the time of the survey were not using ENDS before initiation of the HTP and ultimately transitioned to ENDS exclusive, dual, or poly-use behaviors. The percent of adults who reported current dual use of CC and ENDS (37%, 77 of 209 ENDS users at time of survey) was similar to the level before HTP initiation.

Before use of the HTP, participants smoked an average of 106.3 CPW. CPW decreased to 39.0 during HTP use, while the HTP was on the market, and increased to 76.6 at the time of the survey; each of these changes were significant at the *p* < 0.0001 level. Overall, participants used an average of 65.5 HTP sticks per week before the HTP removal. Those who continued use of the HTP at survey time reported using 70.6 HTP sticks per week before market removal, and this decreased to 43.4 at the time of the survey (*p* < 0.0001).

Table 3 shows results of regression modelling examining the relationship between various tobacco product use behaviors and stopping smoking. HTP and ENDS use were independently associated with higher odds of stopping smoking, while continued smoking of other combustible products was related to smoking at the time of the survey. Compared with non-current use of HTP, adults who currently used HTP had 86% higher odds of stopping smoking (adjusted odds ratio [aOR] 1.86; 95% confidence interval [CI], 1.11–3.11). Further, people who were currently using ENDS had 341% higher odds of stopping smoking (aOR: 3.41; 95% CI 2.13–5.47). People aged 21–34 years had 86% higher odds of stopping smoking (aOR: 1.86; 95% CI 1.01–3.41) compared to those aged 35–49 years, with no other significant effects found by age. No significant differences in stopping smoking were observed between gender or race/ethnicity groups in the adjusted model.

**Table 3:** Smoking status proportions and logistic regression results for stopping smoking

	Adults who formerly smoked	Adults who currently smoked	Chi-Sq *p* value	Unadjusted odds ratio (OR)	Adjusted OR (aOR)
HTP current use at the time of the survey					
Yes	43 (11.3%)	57 (15.0%)	**0.012**	**1.83 (1.14, 2.94)**	**1.86 (1.11, 3.11)**
No	82 (21.5%)	199 (52.2%)		Ref	Ref
Current ENDS use at the time of the survey					
Yes	75 (19.7%)	85 (22.3%)	** < 0.0001**	**3.02 (1.94, 4.70)**	**3.41 (2.13, 5.46)**
No	50 (13.1%)	171 (44.9%)		Ref	Ref
Current other non-combustible tobacco use at the time of the survey					
Yes	13 (3.4%)	20 (5.3%)	0.4007	1.37 (0.66, 2.85)	1.38 (0.62, 3.09)
No	112 (29.4%)	236 (61.9%)		Ref	Ref
Gender					
Male	72 (18.9%)	127 (33.3%)	0.1426	1.38 (0.90, 2.12)	1.25 (0.78, 2.02)
Female	53 (13.9%)	129 (33.9%)		Ref	Ref
Age group					
21–34	32 (8.4%)	43 (11.0%)	0.1181	**1.97 (1.12, 3.46)**	**1.86 (1.01, 3.41)**
35–49	51 (13.4%)	132 (34.7%)		Ref	Ref
50–64	34 (8.9%)	69 (18.1%)		1.28 (0.76, 2.15)	1.63 (0.93, 2.86)
65+	8 (2.1%)	13 (3.4%)		1.59 (0.62, 4.07)	2.10 (0.77, 5.72)
Race/ethnicity					
White	88 (23.1%)	199 (52.2%)	0.1977	Ref	Ref
Black/African American	9 (2.4%)	22 (5.8%)		0.93 (0.41, 2.09)	1.08 (0.45, 2.56)
Asian, Indian, PI	13 (3.4%)	15 (3.9%)		1.96 (0.90, 4.29)	1.67 (0.70, 3.96)
Multiracial/Hisp/Other	15 (3.9%)	20 (5.3%)		1.70 (0.83, 3.47)	2.07 (0.94, 4.56)

Other non-combustible tobacco use included smokeless tobacco pouches and nicotine pouches. Smoking status, whether current or former smoker, was defined based on participant’s smoking status at the time of the survey. Bold font signifies a statistically significant difference (*p* value < 0.05) between the groups being compared

Table 4 shows results from a general linear model (GLM) used to examine the relationship between amount smoked per week after HTP was removed and several predictors. The model was assessed for goodness of fit (F = 37.17, *p* < 0.0001, R-Square = 0.47). Predictors included in the model were the same as the stop smoking model and adjusted for the mean-centered baseline number of CPW prior to HTP initiation. The estimated mean number of CPW after HTP removal was 82.9 cigarettes after accounting for covariates. For each cigarette smoked at baseline, participants smoked 0.68 cigarettes on average at the time of the survey (Estimate: 0.677, 95% CI (0.603, 0.750), t = 18.09, *p* < 0.0001). Continued use of HTP and ENDS was associated with reductions in CPW (HTP Estimate: − 18.802, 95% CI (− 33.960, − 3.645), t =  − 2.44, *p* = 0.0152; ENDS Estimate: − 32.908, 95% CI (− 46.214, − 19.603), t =  − 4.86, *p* < 0.0001). No significant differences were found among demographic predictors.

**Table 4:** General linear model estimates predicting CPW after HTP removal

Predictor	Estimate	95% CI	t-value	Pr > t
Intercept	102.78	88.76, 116.79	14.41	< 0.0001
Mean centered CPW before HTP initiation	0.677	0.603, 0.750	18.09	< 0.0001
HTP current use versus non-use	− 18.802	− 33.960, − 3.645	− 2.44	0.0152
ENDS current use versus non-use	− 32.908	− 46.214, − 19.603	− 4.86	< 0.0001
Other non-combustible current use versus non-use	− 0.565	− 25.023, 23.893	− 0.05	0.9638
Male versus female	5.607	− 7.772, 18.987	0.82	0.4106
21–34 years old versus 35–49 years old	− 16.747	− 34.954, 1.460	− 1.81	0.0713
50–64 years old versus 35–49 years old	− 14.357	− 29.720, 1.005	− 1.84	0.0669
65 + years old versus 35–49 years old	− 16.732	− 45.438, 11.974	− 1.15	0.2526
Black/AA versus White	− 13.146	− 38.264, 11.971	− 1.03	0.3042
Asian, Indian, PI versus White	0.639	− 24.538, 25.816	0.05	0.9602
Multiracial, Hispanic, Other versus White	− 14.339	− 38.131, 9.453	− 1.18	0.2369

Italicized groups refer to the reference category. The model estimates cigarettes per week after HTP removal while accounting for cigarettes per week before the HTP initiation, other tobacco use behaviors, gender, age, and race. Other non-combustible tobacco use included smokeless tobacco pouches and nicotine pouches. “Non-use” as a reference category refers to not using that particular tobacco product

Participants answered questions about behaviors they engaged in when they heard HTP were to be removed from the market and future behavioral intentions to use tobacco products, including HTP. Upon hearing that the HTP was to be removed from the market, 45.8% of participants stockpiled the proprietary HTP sticks. Just under a third (31.2%) of participants who were current HTP consumers at survey time did not report stockpiling HTP sticks. When asked about future intentions to use different tobacco product categories or stopping all tobacco use, participants in this sample were most likely to use the HTP if it returned to market (mean = 4.5; 95% CI 4.3–4.6), then somewhat likely to use cigarettes (mean = 3.8; 95% CI 3.7–4.0) and other smokable products (mean = 3.9; 95% CI 3.8–4.1). They were somewhat unlikely to somewhat likely to use ENDS (mean = 3.3; 95% CI 3.1–3.5) and other HTPs (mean = 2.8; 95% CI 2.7–3.0). They were very unlikely to definitely not likely to use a stop smoking aid (mean = 2.3; 95% CI 2.2–2.5), stop using all tobacco (mean = 2.1; 95% CI 2.0–2.2), use ST (mean = 1.7; 95% CI 1.6–1.8), or nicotine pouches (mean = 1.5; 95% CI 1.4–1.6). Additional results on removal related behaviors and intentions are included in Additional file 1.

## Discussion

This is the first study that addresses changes in intentions and behavior following the introduction and abrupt removal of a tobacco product with a modified risk granted order from the U.S. FDA. The survey design allowed for a retrospective view on tobacco use behavior before, during, and after the HTP was available for purchase in the U.S. among an engaged group of consumers. These results support prior conclusions that HTP and ENDS use among adults who smoke is associated with smoking reductions or stopping smoking [26–28]. The study also identified a substantial portion of individuals who continued to consume the HTP and would prefer to do so in the future.

One prominent phenomenon observed in the study was the significant reduction in smoking prevalence upon introduction of the HTP in the surveyed population. This aligns with other research showing the ability for ENDS and HTP to replace cigarettes [26–28]. A novel finding is that upon removal of the HTP from the market, smoking prevalence did not return to the amount before HTP initiation; instead, there were non-significant increases in smoking prevalence and significant increases in CPW among those who reported smoking. We observed significant increases in ENDS use and continued use of the HTP after removal of the HTP, despite no market availability in the U.S.

Reported use of the HTP even after the market removal order suggests continued purchase of proprietary HTP sticks, potentially through illicit means. While HTP stick stockpiling was reported in almost half of the participants, stockpiling does not likely account for all continued HTP use at the time of the survey. About a third of participants who were still using HTP sticks at the time of the survey did not report stockpiling. Additionally, the 9–10 months between the removal from market and time of the survey would likely lead to exhaustion of all but the most extreme stockpiles. Instead, limited evidence from other surveys indicate that it is common to easily and economically purchase HTP sticks via the internet with international delivery.^3^ While possible to continue to acquire HTP sticks via the mail, the reduction in reported HTP stick packs per week (from 3 to 4 while HTP sticks were available for sale in the U.S. to 2–3 at the time of the survey) suggest that online delivery is not convenient enough to sustain a desired supply. This could be a reason for the lower impact of continued HTP use on reduction of cigarettes at time of survey, in comparison to a large impact while in market as shown in the regression modelling.

The findings from our logistic and GLM regressions indicate that ENDS and HTP use are associated with both stopping smoking and decreases in amount smoked per week. The independent associations of ENDS and HTP with stopping smoking in adjusted logistic regression models may suggest that each of these products had a distinct effect on stopping smoking. Further, some adults poly-used HTP and ENDS post-HTP removal from market, indicating interest in multiple product categories other than cigarettes. In GLM regressions, there was less than one cigarette increase in cigarettes per week among those who continued or returned to smoking compared to use at baseline, whereas there was cigarette reduction equivalent to a pack or more per week among those who were using ENDS or HTP. The concordance in findings from logistic and GLM regressions indicate strong relationship between use of ENDS or HTP and stopping smoking or reductions in CPW.

Increased ENDS use and the lack of a complete return to smoking after the HTP was removed from market was unanticipated. Tobacco harm reduction-related behavior changes like these could be informed by behavior theories such as the Health Belief Model (HBM) or Ecological Systems Theory (EST). Key parts of these theories suggest that behavior is impacted by an interplay of internal and external factors. Internal processes of change (e.g., consciousness raising, self-reevaluation, and stimulus control), self-efficacy and decisional balance are influential social-cognitive variables. Self-efficacy is the confidence that a person can have success in making a desired change [29] and has been associated with progression from contemplation to action stages of behavior change such as smoking cessation [30]. Consequently, improving a person’s self-efficacy can promote behavior change and it could be that making a switch to the HTPs (with their relatively similar form and flavor to cigarettes and with modified risk claims) led to increased belief that stopping smoking could be achieved through quitting or through transition to another potentially reduced harm product like ENDS. While the HBM can be used to explain certain aspects of these behaviors, a socio-ecological model such as EST can help explain the dynamic relationship between personal and external environmental factors [31–33]. The external environment (e.g., social stigma, health care advised cessation/THR advice, FDA position on THR) can have an immense impact on behaviors. Future research should explore health behavior theories’ application to THR strategies.

This study has several limitations to consider when interpreting results. This study includes self-reported behaviors among a sample of adults identified from a comprehensive database of registrants (i.e., 70% of purchasers). These registrants self-selected HTP use and are likely biased towards liking the product. Thus, this sample should be considered to represent people who purchased, used, and possibly preferred the HTP. While these results could in part be applied to users of other reduced harm products, the potential for selection bias can influence representativeness of results (e.g., intentions, switching to ENDS, illicit HTP purchase). While participants are likely to recall general tobacco use transitions, this study used a cross-sectional design to recall three variable time periods including before HTP initiation, during HTP use, and after the HTP was removed from the market; this approach could lead to recall or other biases. The sample of smokeless tobacco, nicotine pouch, and other smokable product categories was small and could impact precision for those estimates. Unknown motivations or beliefs could have impacted behaviors and causality cannot be inferred.

The results of our study suggest the importance of multiple reduced harm product options in the marketplace. The combined and individual impact of HTP and ENDS products in this study sample indicate that adults have differing preferences. This sample of adults with a smoking history who used HTP reported a preference for inhalable products over oral products such as nicotine pouches. Without a satisfactory replacement, adults who smoke may use potentially reduced harm options as at best a temporary replacement [34]. The regression results indicate a significantly higher likelihood of both stopping and reducing smoking among adults who used ENDS and who also at some point used the HTP.

Mounting evidence, including these study results, demonstrates reduction or elimination of combustible cigarette use associated with use of potential reduced harm products such as HTP and ENDS products. More reduced harm products on the market with adequate education on harm relative to cigarette use would likely further reduce smoking among adults who cannot or will not quit tobacco or nicotine-containing product use. Conversely, removing products from the market contributes to increased relative harm as smoking increases and today’s adults who use cigarettes are refused the opportunity for harm reduction. Lastly, demand for products remains even after market removal, potentially leading consumers to illicit products. These study outcomes and observations reveal the opportunity to reduce tobacco-related harm through acceptable reduced harm replacements for combustible cigarettes.

### Supplementary Information

**Additional file 1:** Additional results on behaviors upon learning of the HTP market removal and intentions to use tobacco and nicotine products into the future.

## Data Availability

The data set and materials are available, with some restrictions, via a data request process. To submit a request, please contact the corresponding author.

## Footnotes

1 The claim reads: “AVAILABLE EVIDENCE TO DATE:

- The IQOS^®^ system heats tobacco but does not burn it.
- This significantly reduces the production of harmful and potentially harmful chemicals.
- Scientific studies have shown that switching completely from conventional cigarettes to the IQOS^®^ system significantly reduces your body’s exposure to harmful or potentially harmful chemicals”.

2 Unpublished results from Altria Client Services LLC, CMII Tobacco Product Portfolio Study Phase 1, 2021.

3 Internal review of ALCS Adult Tobacco Consumer Survey interviews.
