# Correction: A reduced exposure heated tobacco product was introduced then abruptly taken off United States shelves: results from a tobacco harm reduction natural experiment

**DOI:** 10.1186/s12954-024-01016-8

**Published:** 2024-06-06

**Authors:** Brendan Noggle, Kevin M. Ball, Andrea Rae Vansickel

**Affiliations:** Center for Research and Technology, Altria Client Services LLC, 601 East Jackson Street, Richmond, VA, 23219 USA

**Harm Reduction Journal (2024) 21:84.**

10.1186/s12954-024-01000-2.

Following the publication of the original article [1], the author’s name, Brendan Noggle, inadvertently appeared twice in the author group. The author group should appear as mentioned below:

Brendan Noggle^1^^*^, Kevin M. Ball^1^ and Andrea Rae Vansickel^1^.

The original article has been corrected.
