# Supplementary material for: A reduced exposure heated tobacco product was introduced then abruptly taken off United States shelves: results from a tobacco harm reduction natural experiment
Source: Harm Reduct J. 2024 Apr 24;21:84. doi: 10.1186/s12954-024-01000-2 (PMC11040862; doi:10.1186/s12954-024-01000-2)

**Additional File 1**

**Reported behavior upon learning of the HTP market removal**

Participants were asked to report their tobacco and nicotine product use behaviors once the HTP was removed from the market with a check all that apply question. Response options included whether they stockpiled or saved HTP sticks for future use, what they did with the HTP device, and what other tobacco and nicotine products, if any, they used to replace their use of the HTP. Supplemental Table 1 presents results for each of these behaviors.

Over half of the participants saved HTP sticks for future use (55.6%; n=279) – some saved HTP sticks through stockpiling only (33.9%; n=170), through rationing only (9.7%; n=49), or by both stockpiling and rationing (12.0%; n=60). Other participants returned the HTP device (10.0%; n=50), then switched to or continued using other tobacco and nicotine products (49.4%; n=248). Few participants began using nicotine replacement therapies (2.6%; n=13).

Supplemental Table 1. Participant action upon HTP market removal

| **Action Upon Market Removal** | **n** | **%** |
| --- | --- | --- |
| Saved HTP Sticks | 279 | 55.6% |
| Stockpiled HTP Sticks Only | 170 | 33.9% |
| Rationed HTP Sticks Only | 49 | 9.7% |
| Stockpiled & Rationed HTP Sticks | 60 | 12.0% |
| Returned HTP Device | 50 | 10.0% |
| Used Other Tobacco and Nicotine Products | 248 | 49.4% |
| Used Nicotine Replacement Therapies | 13 | 2.6% |
| Something Else | 54 | 10.8% |

Most current HTP users had stockpiled HTP sticks (n=86, 68.8%), but others (n=39, 31.2%) did not. Supplemental Table 2 presents the results of proportions of current or former HTP use and HTP stockpiling. Percentages may differ slightly between Supplemental Table 1 and 2 due to rounding.

Supplemental Table 2. Participant stockpiling behaviors by current and former HTP use status with percentages as a proportion of total respondents.

|  | | **Use of the HTP** | | |
| --- | --- | --- | --- | --- |
|  |  | **Current** | **Former** | **Total** |
| **Reported behavior upon learning of market removal** | **Did not stockpile HTP sticks** | 39 (7.8%) | 233 (46.4%) | 272 (54.2%) |
|  | **Stockpiled HTP sticks** | 86 (17.1%) | 144 (28.7%) | 230 (45.8%) |
|  | **Total** | 125 (24.9%) | 377 (75.1%) | 502 (100%) |

**Likelihood of tobacco and nicotine product use into the future**

Participants rated how likely they are to use various tobacco and nicotine products in the future, including the HTP if it returned to market on a scale that ranged from 1 (definitely not likely) to 6 (definitely likely). These results are presented as average likelihood of use ratings in Supplemental Table 3 and Supplemental Figure 1.

Likelihood of use was highest for the HTP among the overall sample. No other tobacco and nicotine product had average likelihood of use ratings above 4.0 (somewhat likely). Likelihood of use ratings for the other tobacco and nicotine products ranged from an average of 1.5 (definitely not likely to very unlikely) for nicotine pouch to 3.8 and 3.9 (somewhat likely) for cigarette and non-cigarette smokable tobacco products, respectively.

Supplemental Table 3. Likelihood of tobacco and nicotine product use into the future

| **Tobacco and nicotine product intention to use** | **Mean score** | **95% Confidence interval** |
| --- | --- | --- |
| The HTP | 4.5 | 4.3 - 4.6 |
| Other Non-Cigarette Smokable Tobacco | 3.9 | 3.8 - 4.1 |
| Combustible Cigarettes | 3.8 | 3.7 - 4.0 |
| Electronic Nicotine Delivery Systems | 3.3 | 3.1 - 3.5 |
| Other HTP | 2.8 | 2.7 - 3.0 |
| Nicotine Replacement Therapies | 2.3 | 2.2 - 2.5 |
| Stop Using All Tobacco Products | 2.1 | 2.0 - 2.2 |
| Smokeless Tobacco | 1.7 | 1.6 - 1.8 |
| Nicotine Pouches | 1.5 | 1.4 - 1.6 |

Supplemental Figure 1. Visualization of mean likelihood of use ratings. ST= smokeless tobacco, ENDS= Electronic Nicotine Delivery Systems.


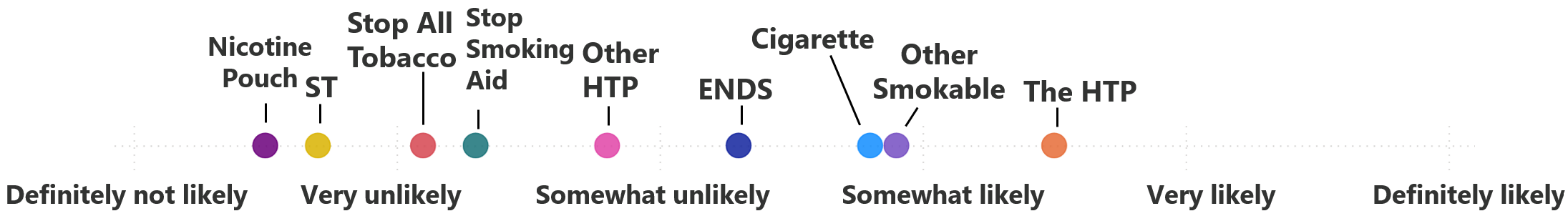

Supplement: Supplementary file 1 — Additional file 1: Additional results on behaviors upon learning of the HTP market removal and intentions to use tobacco and nicotine products into the future. [file 12954_2024_1000_MOESM1_ESM.docx]
